# Virus Hijacks Host Proteins and Machinery for Assembly and Budding, with HIV-1 as an Example

**DOI:** 10.3390/v14071528

**Published:** 2022-07-13

**Authors:** Chih-Yen Lin, Aspiro Nayim Urbina, Wen-Hung Wang, Arunee Thitithanyanont, Sheng-Fan Wang

**Affiliations:** 1 Department of Medical Laboratory Science and Biotechnology, Kaohsiung Medical University, Kaohsiung 80708, Taiwan; pigpipi831205@gmail.com; 2 Center for Tropical Medicine and Infectious Disease, Kaohsiung Medical University, Kaohsiung 80708, Taiwan; aspiro.urbina@hotmail.com (A.N.U.); bole0918@gmail.com (W.-H.W.); 3 Division of Infectious Disease, Department of Internal Medicine, Kaohsiung Medical University Hospital, Kaohsiung Medical University, Kaohsiung 80708, Taiwan; 4 Department of Microbiology, Faculty of Science, Mahidol University, Bangkok 10400, Thailand; arunee.thi@mahidol.ac.th; 5 Department of Medical Research, Kaohsiung Medical University Hospital, Kaohsiung Medical University, Kaohsiung 80708, Taiwan

**Keywords:** assembly, budding, HIV-1, ESCRT, late domain, Alix, galectin-3

## Abstract

Viral assembly and budding are the final steps and key determinants of the virus life cycle and are regulated by virus–host interaction. Several viruses are known to use their late assembly (L) domains to hijack host machinery and cellular adaptors to be used for the requirement of virus replication. The L domains are highly conserved short sequences whose mutation or deletion may lead to the accumulation of immature virions at the plasma membrane. The L domains were firstly identified within retroviral Gag polyprotein and later detected in structural proteins of many other enveloped RNA viruses. Here, we used HIV-1 as an example to describe how the HIV-1 virus hijacks ESCRT membrane fission machinery to facilitate virion assembly and release. We also introduce galectin-3, a chimera type of the galectin family that is up-regulated by HIV-1 during infection and further used to promote HIV-1 assembly and budding via the stabilization of Alix–Gag interaction. It is worth further dissecting the details and finetuning the regulatory mechanism, as well as identifying novel candidates involved in this final step of replication cycle.

Viruses are nanoscale entities containing a nucleic acid genome encased in a protein shell called a capsid and in some cases surrounded by a lipid bilayer membrane. The formation of a virus is a remarkable feat of natural engineering [1]. A variety of protein subunits and other components assemble from the crowded cellular milieu to form reproducible structures on a biologically relevant time scale. The last step of the virus life cycle is assembly and budding, which is the key determinant of virus replication [1,2]. During this phase, the newly synthesized viral genome and proteins are assembled to form new virus particles, which then exit the host cell and acquire a host-derived membrane enriched in viral proteins to form their external envelope. Viral assembly may take place in the cell nucleus, cytoplasm, or plasma membrane, whereas viral budding can occur at every stage in the ER–Golgi–cell membrane pathway depending on virus type [2,3,4,5]. It is known that both viral and host proteins are required in this process. A better understanding of this virus–host protein interaction is essential to gain fundamental insights into the functions and properties of these proteins and further develop novel anti-viral strategies.

It is known that several enveloped viruses bud through membranes where they acquire the lipid bilayers by employing the ubiquitous strategy of appropriating the cellular ESCRT (endosomal sorting complexes required for transport) pathway [6] Figure 1. This approach is particularly useful since ESCRT pathways are conserved across eukaryotes and certain archaea. Retroviruses, especially HIV-1, take advantage of this pathway to bud out of cells. ESCRT machinery is required for the multivesicular body (MVB) pathway and cytokinesis, which contain five different complexes (ESCRT-0, -I, -II, -III, and Vps4) that have distinct functions [7]. The early acting ESCRT complexes (ESCRT-I and ESCRT-II) assemble stably within the cytoplasm and are associated with adaptor proteins (such as the HRS/STAM complex, also named ESCRT-0) to recruit and activate late-acting ESCRT-III and VSP4 factors at specific membrane sites where virion assembly and fission events occur [7,8,9,10]. Viruses are known to hijack ESCRT complexes and some adaptor proteins to facilitate this final step via their “late” (L) domain motifs [11]. Historically, L domains were first identified within Gag structural precursors of retroviruses [12] Figure 1A. Currently, L domains are found in structural proteins of many RNA enveloped viruses, such as arenaviruses, filoviruses, rhabdoviruses, reoviruses, and paramyxoviruses [11].

Retroviruses encode three main classes of late domains: (1) Pro-Thr/Ser-Ala-Pro (PT/SAP) motifs, which can interact with Tsg101 (a component of ESCRT-I); (2) Tyr-Pro-Xn-Leu (YPXnL) motifs, where X is a variable residue and n is 1–3 that can bind the apoptosis-linked gene 2 (ALG-2)-interacting protein (Alix, formerly known as AIP1), a protein that harbors binding sites for both ESCRT-I and ESCRT-III; and (3) Pro-Pro-Pro-Tyr (PPPY) motifs, which interact with members of the Nedd4 family of E3 ubiquitin ligases [13,14,15,16]. There are several different types of L domains reported in retroviruses, and some well-studied types of L domains are illustrated in Figure 1A. During the late phase of the HIV-1 life cycle, Gag polypeptides and the viral accessory protein, Vif, are associated with a host protein HP68. HP68, an ATP binding protein, appears to interact with the NC region of Gag and promotes the progression of Gag-containing assembly intermediates into immature capsids at the host cell plasma membrane [17,18]. Furthermore, p6 late domain would hijack Alix and Tsg101 to recruit the ESCRT complex for the facilitation of virus budding (Figure 1B).

Alix is composed of three major structural domains: an N-terminal domain, which interacts with ESCRT-III component charged multivesicular body protein 4 (CHMP4); a V domain, which binds YPXnL motifs of HIV-1 and EIAV Gag; and a proline-rich domain (PRR), which binds a number of factors (including the ESCRT-I component Tsg101) [19] (Figure 1B). Apart from MVB biogenesis, Alix also acts in apoptosis, endocytosis, and cytokinesis pathways. Alix and Tsg101 are two major ESCRT complex-related adaptor proteins that are hijacked and utilized by p6 subunit of HIV-1 Gag. In addition, Vps28 binds to Tsg101 and appears to be essential for budding. Although p6 is a small protein, it is encoded by one of the most polymorphic regions of the HIV-1 gag gene and undergoes numerous posttranslational modifications such as ubiquitination, phosphorylation, and SUMOylation [20]. Further, p6 also mediates accessory protein Vpr into budding HIV-1 virions. Recent studies demonstrate that the mutation or truncation occurring in Alix- or Tsg101-binding domain on p6 significantly abrogated HIV-1 budding [19,21]. The primary Alix binding motif is located near the C-terminus of p6, between residues 36 and 44 (^36^YPLASLRSL^44^). The site Y36, L41, and L44 of p6 are critical for Gag–Alix binding [22]. In addition, the NC domain of HIV-1 p6 can bind with Alix, suggesting that this domain provides alternative links between Gag and ESCRT-I and ESCRT-III. The p6 N-terminus contains a PT/SAP motif, which interacts with Tsg101, playing a major role in HIV-1 budding [22]. The fusion of Tsg101 to the C-terminus of HIV-1 p6 rescues the PT/SAP mutation-mediated budding defect, while the overexpression of the N-terminal blocks HIV-1 budding in a dominant-negative manner [21,23]. The overexpression of full-length Alix or its N-terminal Bro1 domain rescues the defect in particle budding imposed by the mutation of the PT/SAP motif [24,25]. Stabilized interactions between Alix or Tsg101 and p6 Gag are beneficial to HIV-1 replication.

Furthermore, in addition to the recruitment of the ESCRT complex, Gag protein play a predominant role in guiding several events, such as protein–protein interactions necessary to create spherical particles and the concentration of the viral Env protein, binding to the plasma membrane, and assisting in the genomic RNA package and multimerization, leading to membrane curvature and budding [26,27]. Recent studies have reported that the occurrence of structural transition between the immature Gag lattice and the formation of the mature viral capsid core are key features of HIV-1 assembly and maturation [28]. During or shortly following budding, the HIV protease (PR) immediately activates and cleaves the immature Gag precursor into different subunit components, including matrix (MA), capsid (CA), spacer peptide 1 (SP1), nucleocapsid (NC), spacer peptide 2 (SP2), and p6 [27,28]. However, a recent study indicated that PR becomes activated during assembly and budding prior to particle release [29]. Gag oligomerization is known to trigger virus assembly. The viral genome is recruited by Gag and then directed and anchored to the plasma membrane. MA–membrane binding and NC–RNA interaction play functionally redundant roles to promote Gag oligomerization [30,31]. Viral RNA and plasma membranes are proposed as scaffolds for Gag multimerization. In addition, fatty acid modification on the N-terminus of MA via myristoylation is also critical to the HIV assembly process [32,33]. Mutations of the N-terminal glycine residue of MA affect myristylation and further cause severe defects in virus assembly. In addition, a recent fundamental study indicated that inositol hexakisphosphate (IP6) (a small polyanion also known as phytic acid) plays a role in the formation of both the immature Gag lattice and the mature capsid, suggesting that IP6 stabilizes the immature Gag lattice and is a major determinant of HIV-1 assembly [34,35].

Recently, some cellular proteins have been reported to facilitate HIV virus replication, for example, galectin-3 (Gal3), a chimera type of the galectin family, exerting both endogenous and exogenous regulatory capabilities to various cellular immunol functions and even infectious status [36,37] (Figure 1B). The up-regulation of Gal3 was reported during HIV-1 infection by the Tat protein binding to the Gal3 expression promoter [38]. However, it is still not fully understood why these S-type lectins are induced by HIV-1 infection. A recent work indicated that HIV-1 infection triggered Gal3 induction, which would interact with Alix, subsequently stabilizing Alix–p6 Gag interaction, suggesting that Gal3 is an alternative adaptor protein that could be used by HIV-1 virus for assembly and budding [36]. When HIV-1 p6 Gag has truncations in the Alix-binding domain (such as certain HIV-1 CRF07_BC isolate), it leads to a reduction in Alix binding, subsequently influencing new virus release [39]. More recently, Okamoto et al., reported that Gal3 expression is closely correlated with HIV-1 expression in latently infected cells through NF-κB activation and the interaction with Tat, implying another role of Gal3 in HIV-1 infection [40].

Although the step and mechanism regarding HIV-1 assembly and budding have been well-elucidated, there are many new proteins being identified that participate in the regulation of the HIV-1 replication cycle. It is worth identifying novel candidates involved in assembly and budding and further understanding their interaction with ESCRT complexes or adaptor proteins during HIV-1 infection.

## Figures and Tables

**Figure 1:**
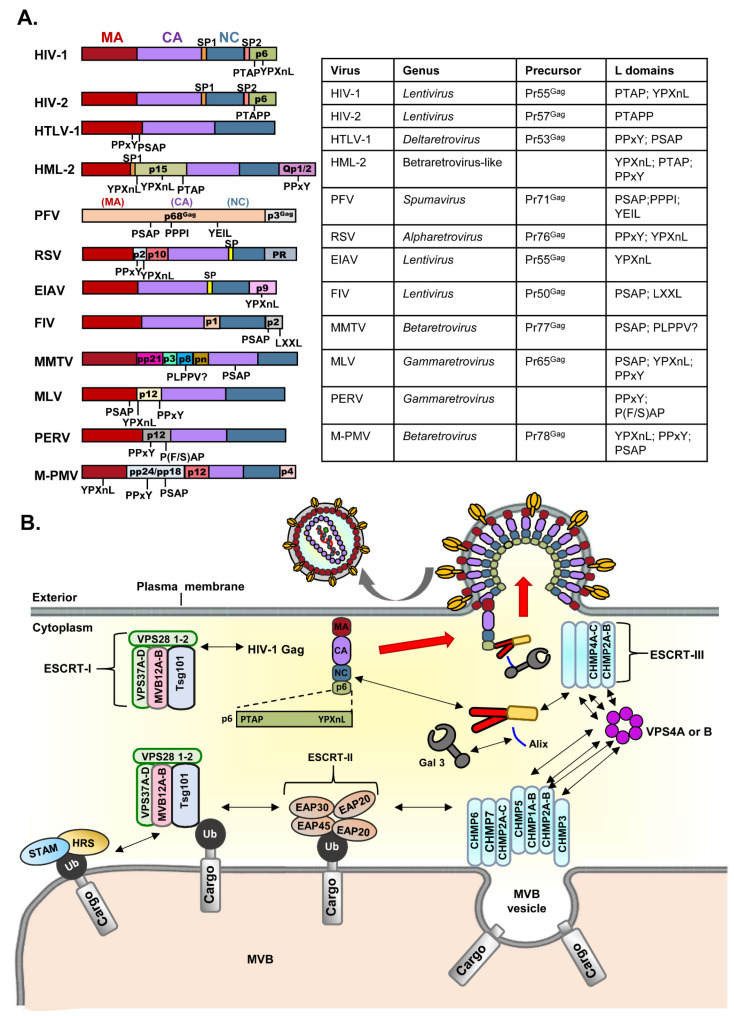
HIV-1 Gag precursor containing late domains that hijack the host Endosomal Sorting Complex Required for Transport (ESCRT) machinery components and cellular adaptors to facilitate virus assembly and budding. (**A**) The peptide sequences containing L domains, which are indicated within structural precursors of several retroviruses, including Human Immunodeficiency Virus type 1 (HIV-1), Human Immunodeficiency Virus type 2 (HIV-2), Human T Cell Leukemia Virus type 1 (HTLV-1), Human Endogenous Retrovirus-K (HML-2), Prototypic Foamy Viruses (PFV), Rous Sarcoma Virus (RSV), Equine Infectious Anemia Virus (EIAV), Feline Immunodeficiency Virus(FIV), Mouse Mammary Tumor Virus (MMTV), Murine Leukemia Virus (MLV), Porcine Endogenous Retrovirus (PERV)m and Mason–Pfizer Monkey Virus (M-PMV) are illustrated. (**B**) A schematic representation of late domains hijacking ESCRT machinery components and cellular adaptors in the HIV-1 replication cycle is shown. The lower image indicates the formation of multivesicular bodies’ (MVBs’) vesicles on late endosomes that contain cargo destined for lysosomal degradation. MVB formation requires the activity of ESCRT complexes I, II, and III, which are sequentially or concentrically recruited to the endosomal membrane to sequester cargo proteins and drive vascularization into the endosome to regulate the vacuolar protein sorting pathway as well as the formation of vesicles that bud away from the cytoplasm. The upper image indicates HIV-1 Gag containing L domains that can hijack the ESCRT complex, which was originally used for MVB biogenesis for viral assembly and budding (Vacuolar protein sorting-associated protein (VPS), Multivesicular Body (MVB); Tumor susceptibility gene 101 (Tsg101); Charged multivesicular body protein (CHMP); Galectin-3 (Gal3); Expanded access program (EAP); Ubiquitin (Ub); Hepatocyte growth factor-regulated tyrosine kinase substrate (HRS); Signal transducing adaptor molecule (STAM)).

## Data Availability

The data that support the findings of this study are available upon request from the authors.
